# Efficacy and safety of clonidine for the treatment of impulse control disorder in Parkinson’s disease: a multicenter, parallel, randomised, double-blind, Phase 2b Clinical trial

**DOI:** 10.1007/s00415-023-11814-y

**Published:** 2023-06-20

**Authors:** Chloé Laurencin, Noémie Timestit, Ana Marques, Domitille Dilly Duchez, Caroline Giordana, Sara Meoni, Marine Huddlestone, Teodor Danaila, Mathieu Anheim, Hélène Klinger, Tiphaine Vidal, Marion Fatisson, Catherine Caire, Mikail Nourredine, Philippe Boulinguez, Carole Dhelens, Bénédicte Ballanger, Stéphane Prange, Sylvie Bin, Stéphane Thobois

**Affiliations:** 1 Department of Neurology C, Expert Parkinson Centre, Hospices Civils de Lyon, Pierre Wertheimer Neurological Hospital, Hôpital Neurologique Pierre Wertheimer, Service de Neurologie C - Hospices Civils de Lyon, NS-Park/F-CRIN, 69677 Bron, France; 2 Lyon Neuroscience Research Centre, INSERM, University of Lyon, 69622 Lyon, France; 3 Department of Biostatistics, University Hospital of Lyon, Lyon, France; 4 Department of Neurology, Clermont-Ferrand University Hospital, NS-Park/F-CRIN, Clermont-Ferrand, France; 5 Department of Neurology, University Hospital of Saint-Etienne, Saint-Etienne, France; 6 Department of Neurology, University Hospital of Nice, NS-Park/F-CRIN, Nice, France; 7 Movement Disorders Unit, Department of Neurology, University Hospital of Grenoble, NS-Park/F-CRIN, Grenoble, France; 8 Department of Neurology, Strasbourg University Hospital, Strasbourg, France; 9 Pharmacotoxicology Laboratory, Department of Clinical Research and Epidemiology, University Hospital of Lyon, Lyon, France; 10 Pharmacy, FRIPHARM, Edouard Herriot Hospital, Lyon University Hospital, Lyon, France; 11 Public Health Center, Research and Clinical Epidemiology, University Hospital of Lyon, Lyon, France; 12 Marc Jeannerod Cognitive Neuroscience Institute, CNRS, UMR 5229, Bron, France; 13 Faculté de Medecine Et de Maieutique Lyon Sud Charles Mérieux, Université Claude Bernard Lyon 1, Université de Lyon, Lyon, France; 14 Institut de Génétique Et de Biologie Moléculaire Et Cellulaire (IGBMC), INSERM-U964/CNRS, UMR7104/Strasbourg University, Illkirch, France; 15 Centre de Référence Des Maladies Neurogénétiques Rares, Fédération de Médecine Translationnelle de Strasbourg (FMTS), Université de Strasbourg, Strasbourg, France

**Keywords:** Clonidine, Noradrenergic system, Parkinson’s disease, Impulse control disorder, QUIP-RS

## Abstract

**Background:**

Impulse control disorders (ICDs) are frequently encountered in Parkinson’s disease (PD).

**Objectives:**

We aimed to assess whether clonidine, an α2-adrenergic receptor agonist, would improve ICDs.

**Methods:**

We conducted a multicentre trial in five movement disorder departments. Patients with PD and ICDs (*n* = 41) were enrolled in an 8-week, randomised (1:1), double-blind, placebo-controlled study of clonidine (75 μg twice a day). Randomisation and allocation to the trial group were carried out by a central computer system. The primary outcome was the change at 8 weeks in symptom severity using the Questionnaire for Impulsive-Compulsive Disorders in Parkinson’s Disease–Rating Scale (QUIP-RS) score. A reduction of the most elevated subscore of the QUIP-RS of more than 3 points without any increase in the other QUIP-RS dimension defined success.

**Results:**

Between 15 May 2019 and 10 September 2021, 19 patients in the clonidine group and 20 patients in the placebo group were enrolled. The proportion difference of success in reducing QUIP-RS at 8 weeks, was 7% (one-sided upper 90% CI 27%) with 42.1% of success in the clonidine group and 35.0% in the placebo group. Compared to patients in the placebo group, patients in the clonidine group experienced a greater reduction in the total QUIP-RS score at 8 weeks (11.0 points vs. 3.6).

**Discussion:**

Clonidine was well tolerated but our study was not enough powerful to demonstrate significant superiority compared to placebo in reducing ICDs despite a greater reduction of total QUIP score at 8 weeks. A phase 3 study should be conducted.

**Trial Registration:**

The study was registered (NCT03552068) on clinicaltrials.gov on June 11, 2018.

**Supplementary Information:**

The online version contains supplementary material available at 10.1007/s00415-023-11814-y.

## Introduction

Impulse control disorders (ICDs) are behavioural disorders frequently encountered in Parkinson’s disease (PD) treated by dopamine replacement therapies (DRT). The main ICDs are pathological gambling, binge eating, compulsive shopping, and hypersexuality [1].

A recent study reported a 5‐year cumulative ICD incidence rate of 46% [1]. Risk factors for ICDs are duration and dose of DA treatment, male gender, comorbid apathy, anxiety, depression, and bipolar disorders [2–4]. Genetic factors of susceptibility are also suspected [5]. ICDs are underdiagnosed because patients sometimes do not recognise these disorders and often underestimate their severity [6]. The lack of insight and a defence mechanism (with denial or feeling of shame) are the main explanations reported [6]. ICDs reduce patients’ quality of life and place a heavy burden on their caregivers [7]. No treatment exists for ICDs. The classical approach consists of reducing or discontinuing DA with a risk of dopamine withdrawal syndrome, which associates anxiety, irritability, apathy and worsening of motor symptoms, and concern approximately a third of PD patients [2, 8]. However, in a retrospective study, only 40% of the PD patients with ICDs were in remission following a reduction or discontinuation of DA after 3.5 years of follow-up [9].

The mechanism of ICDs is not entirely understood but dysfunction of the mesocorticolimbic dopaminergic pathway plays a key role and abnormal sensitization of the limbic dopaminergic system, combined with a relatively preserved dopaminergic mesocorticolimbic pathways and reduced dopamine transporter (DAT) expression in ventral striatum have been clearly shown [10]. Beyond the dopaminergic system, glutamatergic and opioidergic neurotransmission dysfunction could also participate in ICD pathophysiology, which led to clinical trials with controversial or negative results. A small clinical trial with amantadine showed positive results on pathological gambling, because it was hypothesized that dyskinesia and ICDs depend on common mechanisms involving alterations of glutamate homeostasis [11]. However, amantadine was also demonstrated to be associated with the development of ICDs [12]. Naltrexone, an opioid receptor antagonist, was also tested in ICDs because of its efficacy in alcohol dependence but the study failed to demonstrate any efficacy [13]. Recently evidence in PD has pointed to early neuronal loss in the locus coeruleus (LC), which is the primary component of the noradrenergic system that is involved in functions such as arousal, behavioural flexibility, executive function, learning, memory, and wakefulness [14]. In addition, noradrenergic dysfunction is involved in some non-motor symptoms of PD such as depression [15] and dysexecutive symptoms [16], but also in proactive inhibition that is disproportionate in PD favouring akinesia [17]. More specifically, clonidine, an α2-adrenergic agonist, was found to cancel the positive action of subthalamic nucleus-deep brain stimulation (STN-DBS) on akinesia, consistent with the role of noradrenalin (NA) in movement initiation via the modulation of α2-adrenoceptors and inhibitory control [18]. Many effects of clonidine have been attributed to presynaptic α2-adrenoreceptors, resulting in the inhibition of neural release of NA, however, higher doses of clonidine may preferentially [19, 20] engage post-synaptic receptors, enhancing noradrenergic transmission [15]; these effects may have an impact on the top-down control role of the prefrontal cortex [21].

There is also evidence of a role of the noradrenergic system in decision-making and reward processes in rodents [21]. In humans, α2-adrenergic antagonists, such as yohimbine, raise impulsivity [22]. Furthermore, atomoxetine, a NA re-uptake inhibitor, may reduce motor impulsivity in PD [23]. In heroin addicts, a randomised trial found that clonidine improved decision-making performance as assessed by the Iowa Gambling Test and decreased impulsivity [24]. Furthermore, clonidine is commonly used in attention deficit hyperactivity disorder and Tourette’s syndrome and provides benefits on hyperactivity and impulsivity [25]. Based on these results it would be of great interest to analyse the impact of clonidine on ICDs in PD, which has never been done so far.

In this context, we conducted a multicentre prospective, randomised, double-blind placebo-controlled trial to investigate the efficacy of clonidine on ICDs and its safety in PD patients in a phase 2b study in preparation for a phase 3 trial.

## Patients and methods

### Patients

Patients were screened in five university hospital movement disorder departments in France (Lyon, Clermont-Ferrand, Saint Etienne, Grenoble, and Nice). We included patients diagnosed with PD at least 1 year previously and in accordance with the Movement Disorder Society criteria, who were aged [30–80] years, and who weighed [40–95] kg. ICD symptoms had to start after PD onset and initiation of DRT. The Questionnaire for Impulsive-Compulsive Disorders in Parkinson’s Disease-Rating Scale (QUIP-RS) was used to assess the presence and severity of ICDs. The QUIP-RS is a screening instrument for ICDs [26]. Its three sections focus on: (i) the four most common ICDs; (ii) punding and hobbyism; and (iii) compulsive medication use; the higher the score the more severe are the ICDs. Inclusion criteria consisted of a QUIP-RS total score ≥ 10 or a sub-score > 5 for pathological gambling,  > 7 for compulsive shopping or hypersexuality, or > 6 for binge eating. All therapeutic classes of antiparkinsonian treatments were allowed, including DA, but had to be stable in the last 2 months and remain stable during the study. Exclusion criteria were depression (Beck Depression Inventory [BDI II] > 19), dementia (Montreal Cognitive Assessment [MOCA] < 20), orthostatic hypotension, renal failure (globular filtration rate < 30 ml/min/1.73m^2^), bradyarrhythmia, heart failure or coronary disease, Raynaud disease, or addiction to a substance defined by the DSM-IV (except tobacco); in addition, patients with a treatment that could interact with the noradrenergic system (see list in supplemental data) were also excluded. Furthermore, based on the judgement of a study investigator (neurologist), patients with severe ICD necessitating immediate tapering of dopaminergic medication were excluded.

Approval from the institutional ethical standards committee on human experimentation (Comité de protection des personnes Ouest IV -Nantes) was obtained before study initiation and the trial was done in accordance with the Declaration of Helsinki and the International Conference on Harmonisation Good Clinical Practice Guidelines. All patients provided written informed consent before randomisation (EudraCT number 2019-000165-20). The study was registered (NCT03552068) on clinicaltrials.gov.

## Methods

Patients received oral clonidine (75 µg twice a day, a morning and an evening) or a placebo for 8 weeks without any titration. Patients were instructed not to change their PD treatment (medications and/or DBS settings) during the study and treatment was checked at each visit. Randomisation and allocation to the trial group were carried out by a central computer system. Assignment was masked from the patients, study staff, investigators, and data analysts. The QUIP-RS was administered by a neuropsychologist specialised in PD. The neuropsychologists were trained before the beginning of the study in order to standardise, between centres, the administration of the questionnaire. When two sub-scores were equal, we the most disabling for the patient according to the neurologist was retained. Depression symptoms were assessed using the BDI II [27]. We studied hyper- and hypodopaminergic mood and behaviour using the Ardouin scale [28], anxiety using the State-Trait-Anxiety Inventory (STAI-Y), and sleepiness using the Epworth scale [29]. The 39-item Parkinson’s Disease Questionnaire (PDQ-39) [30] was used to evaluate quality of life. We assessed the tolerability (general physical examination, electrocardiogram, blood pressure, pulse measurement) and adherence (capsule counts and patient journal) at weeks 2, 4 and 8. Orthostatic hypotension was searched for (measurement of blood pressure after 5 min lying down, and at 1, 3, 5 and 10 min after standing up) at baseline, as well as at 2, 4 and 8 weeks. Adverse events were monitored throughout the study with a specific interest on orthostatic hypotension and related falls that were considered as serious adverse events. A visit was scheduled at 2 weeks after the inclusion visit, in order to check the tolerance and adherence. At 4 and 8 weeks after randomisation, the primary and secondary criteria were collected in addition to the tolerance and adherence parameters. One visit was scheduled one month after the end of the study.

### Primary endpoint

The efficacy of the clonidine after 8 weeks of treatment was determined using the QUIP-RS. Success corresponded to a decrease of more than 3 points of the more elevated sub-score, without elevation of another sub-score of the QUIP-RS. This criterion was chosen to consider the most marked ICD for a given patient, which seemed more clinically relevant.

### Secondary endpoints

Change in ICD severity was assessed by the variation of the total QUIP-RS score between baseline and week 4 and between baseline and week 8. Secondary outcomes were the changes between baseline and week 8 of the Movement Disorder Society Unified Parkinson’s Disease Rating Scale MDS-UPDRS part I (Non-Motor Aspects of Experiences of Daily Living), part II (motor aspects of experiences of daily living), part III (motor symptoms), and part IV (motor complications) scores [31].

Changes in depression symptoms were assessed using the BDI II [27], in hyper- and hypodopaminergic mood and behaviour using the Ardouin scale or ECMP [28], in anxiety with the STAI-Y, and sleepiness with the Epworth scale [32]. The 39-item Parkinson’s Disease Questionnaire (PDQ-39) [30] evaluated the modification of the quality of life [32].

### Statistical analysis

The primary outcome was a reduction of QUIP-RS score between baseline and 8 weeks. We defined a success as a reduction by 3 points or more between baseline and 8 weeks of the highest QUIP-RS subscore at baseline, without any augmentation in the other QUIP-RS dimension. The minimal clinical difference was set at a 15% difference of success between placebo and clonidine. Using a confidence interval (CI) approach [33], we estimated that 19 patients per group will produce a one-sided upper 90% CI, which excludes 15% assuming that the difference between placebo and clonidine is 0% or less. If the one-sided 90% CI contains 15%, a phase 3 RCT could be considered. We performed a post hoc analysis using a linear mixed effect model to test for time by group differences for treatment response, using fixed effect for randomisation group, time and their interaction, and a random intercept and slope by subject as random effects. The primary outcome analysis included all patients who were enrolled (intention to treat [ITT] analysis). We used the maximum bias approach for handling missing values. We imputed a success for the placebo participant missing primary outcome and a failure for the clonidine participant missing primary outcome. We defined the per protocol population (PP population) as all the patients enrolled who completed the primary outcomes measure. The PP population was used only for the calculation of the primary endpoint. No hypothesis test was performed on secondary outcomes to avoid type I error inflation. Statistics were done using SAS (Statistical Analysis Software 9.4, SAS Institute Inc, Cary, NC, USA).

### Role of the funding source

The funding sources had no role in study design, data collection, data analysis, data interpretation, or writing of the report. The corresponding author has full access to all the data and takes final responsibility to submit for publication.

## Results

**Fig. 1:**
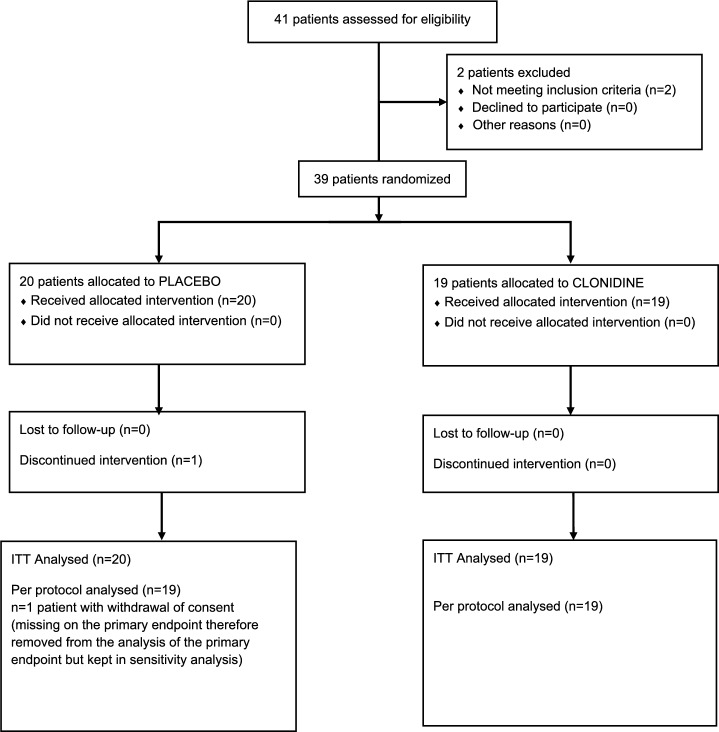
Flow chart

Between 15 May 2019 and 10 September 2021, 41 patients were screened and 39 enrolled and randomly assigned. Nineteen received clonidine and 20 placebos (ITT population). One patient in the placebo arm decided to stop the study because of a personal issue independent of ICD. 19 patients in the clonidine arm and 19 patients in the placebo arm completed the study (PP population; Fig. 1). The groups were balanced in terms of baseline demographic characteristics; the mean (± standard deviation, SD) total QUIP-RS score at baseline was higher in the clonidine arm (28.8 ± 13.2) than in the placebo arm (23.1 ± 7.8; Table 1). In addition to the fulfilling of inclusion and exclusion criteria, no patients had a history of bipolar disorders nor presented mania at inclusion based on DSM V criteria.

**Table 1:** Baseline characteristics

Variable	Placebo	Clonidine
*N*	20	19
Age (years)		
Median (range)	56 (38–75)	61 (45–72)
Mean (SD)	56.3 (9.3)	59.0 (8.2)
Male sex		
* N* (%)	13 (65)	15 (79)
Time since diagnosis (years)		
Median (min—max)	6 (2–19)	9 (2–17)
Mean (SD)	7.3 (4.7)	8.7 (4.9)
QUIP-RS total score		
Median (min–max)	23 (10–39)	27 (10–62)
Mean (SD)	23.1 (7.8)	28.8 (13.2)
Highest QUIP-RS sub-score at baseline *N* (%)		
Pathological gambling	2 (10⋅0)	1 (5⋅3)
Hypersexuality	8 (40.0)	5 (26.3)
Compulsive buying	0	1 (5.3)
Eating disorder	6 (30)	6 (31.6)
Hobbyism	4 (20)	3 (15.8)
Punding	0 (0)	3 (15.8)
Dopamine dysregulation syndrome	0 (0)	0 (0)
Total levodopa equivalent daily dosage (mg/day)		
Median (min—max)	830 (250–1808)	889 (100–2500)
Mean (SD)	946.6 (458.9)	1076.1 (655.2)
Agonist dopaminergic, (% yes)		
* N* (%)	20 (100)	15 (78.9)
MDS-UPDRS 1: (/52)		
Median (min–max)	14.5 (9–22)	14.0 (5–27)
Mean (SD)	14.2 (3.9)	14.6 (6.5)
MDS-UPDRS 2: (/52)		
Median (min–max)	9 (4–2)	11 (3–3)
Mean (SD)	11.3 (6.2)	13.0 (7.6)
MDS-UPDRS 3: (/132)		
Median (min–max)	15.5 (4–50)	17.0 (8–48)
Mean (SD)	18.9 (12⋅0)	20.5 (10⋅3)
MDS-UPDRS 4: (/24)		
Median (min–max)	4 (0–9)	4 (0–13)
Mean (SD)	3.6 (3.0)	5.21 (4.2)
MDS-UPDRS total score: ( /260)		
Median (min–max)	50 (25–80)	44 (27–102)
Mean (SD)	48.8 (16.8)	53.3 (22.1)

### Primary endpoint

The difference of success rate in reducing QUIP-RS at 8 weeks, was of 7% between groups (one-sided upper 90% CI 27%), with 8/19 patients experiencing success in the clonidine group (42.1%) and 7/20 in the placebo group (35.0%) in the ITT population. In the PP population, the proportion difference was of 10% (one-sided upper 90% CI 30.5) with 42.1% of success in the clonidine group and 31.6% in the placebo group.

### Secondary endpoints

**Fig. 2:**
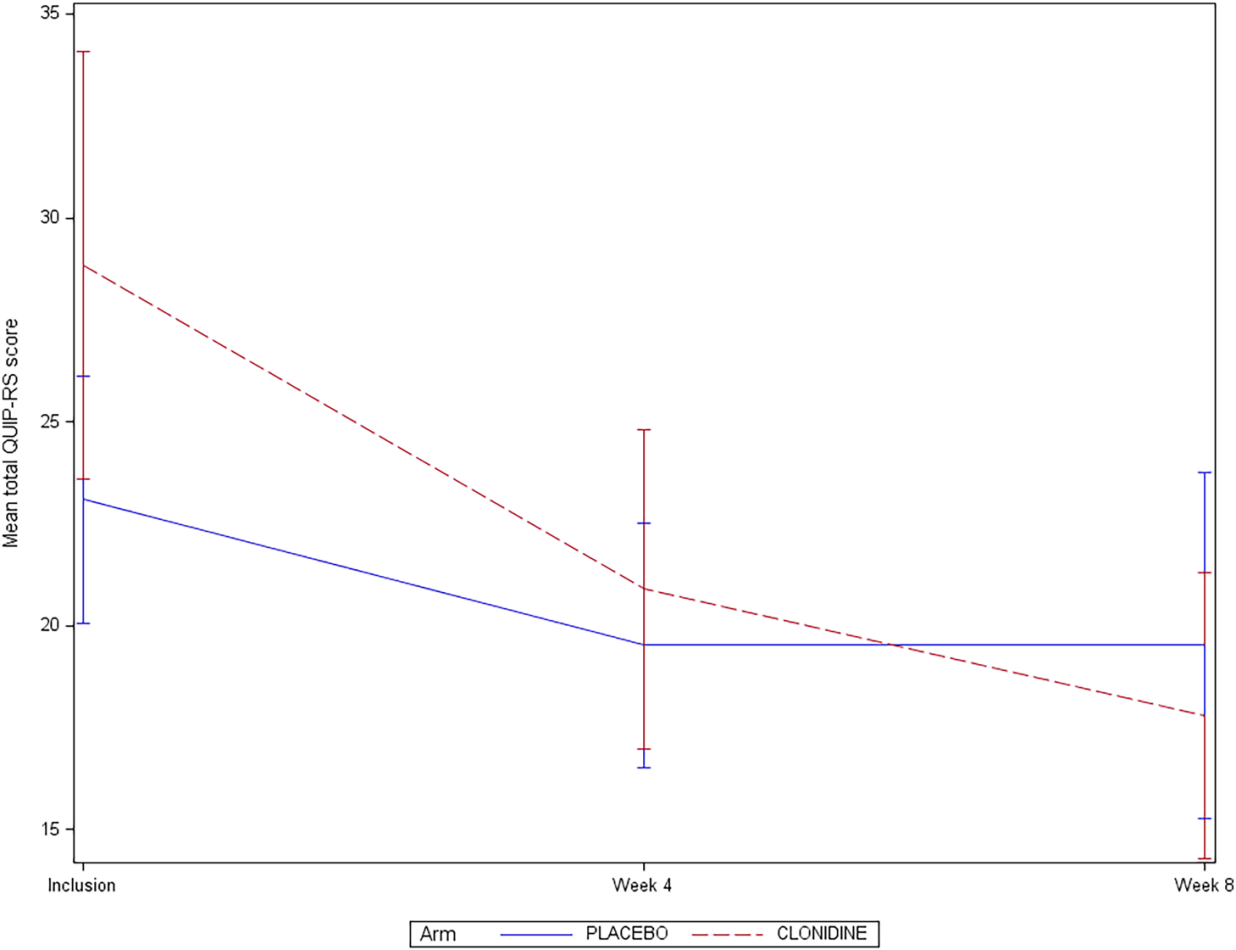
QUIP-RS total score at baseline, 4 and 8 weeks for placebo and clonidine group

At 4 weeks, compared to patients in the placebo arm, patients receiving clonidine had a larger reduction of the mean total QUIP-RS score (7.9 versus 2.9 points). At 8 weeks, this difference became greater; 11.0 points under clonidine versus 3.6 under placebo (Fig. 2).

The frequency of success in reducing QUIP-RS at 4 weeks did not differ between groups (4/20 in the placebo arm (20%) and 4/19 in the clonidine arm (21%).

In the mixed effect model, the estimated mean difference of QUIP-RS evolution between clonidine and placebo groups was  – 3.8 (95%CI [ – 7.2; 0.4]) (see details in supplemental data).

There was no worsening of motor or non-motor manifestations under clonidine.

The MDS UPDRS total and component scores, STAI-Y-A and B, ECMP, and the Epworth scale remained stable at 8 weeks in the ITT population. The PDQ39 score was better at 8 weeks than inclusion in the clonidine group and worse in the placebo group (Table 2).

**Table 2:** Secondary objectives

	Inclusion		Week 8	
	Placebo (*N* = 20)	Clonidine (*N* = 19)	Placebo (*N* = 20)	Clonidine (*N* = 19)
QUIP-RS	23.10 (7.8)	28.8 (13.2)	19.5 (10.7)	17.8 (8.8)
UPDRS total score (/260) Mean (SD)	48.8 (16.9)	53.3 (22.1)	48.9 (21.9)	52.6 (21.2)
UPDRS I (/52) Mean (SD)	14.2 (3.9)	14.6 (6.5)	12.5 (5.3)	13.8 (6.7)
UPDRS II (/52) Mean (SD)	11.3 (6.2)	12.9 (7.6)	12.7 (7.9)	12.8 (8.0)
UPDRS III (/132) Mean (SD)	18.9 (12.1)	20.5 (10.3)	19.6 (11.6)	19.4 (11.1)
UPDRS IV (/24) Mean (SD)	3.6 (3.0)	5.2 (4.2)	3.7 (4.0)	4.1 (3.8)
BDI II (/63) Mean (SD)	8.8 (5.7)	9.2 (5.3)	7.1 (5.8)	8.7 (8.6)
STAI-Y-A (State) (/80) Mean (SD)	28.8 (10.3)	27.7 (11.0)	28.4 (11.9)	27.3 (9.0)
STAI-Y-B (Trait) (/80) Mean (SD)	43.6 (10.5)	38.8 (10.1)	37.4 (11.2)	36.7 (9.9)
PDQ-39 (/100) Mean (SD)	40.3 (19.5)	41.7 (23.9)	48.7 (20.9)	36.5 (31.0)
Epworth (/24) Mean (SD)	11.7 (5.1)	9.3 (4.4)	13.5 (5.4)	8.9 (5.0)
ECMP I Hypodopaminergic disorders Median (range)	4.0 (3, 6)	4.0 (2, 7)	3.5 (2, 6)	3.0 (2, 5)
ECMP II Non motor fluctuations Median (range)	1.5 (0, 3.5)	2.0 (0, 4)	1.5 (0, 3)	1.0 (0, 4)
ECMP II Hyperdopaminergic disorders Median (range)	12.5 (6, 17)	12.0 (9, 12)	7.5 (4, 11)	7.0 +

### Adherence

Patients in the clonidine group had a good adherence monitored using capsule count; 74% of the patients received at least 75% of treatment doses for all periods between visits.

### Safety

Overall, clonidine was well tolerated with no unexpected safety issues (Table 3). During the study, there were three positive orthostatic hypotension tests in the clonidine arm and two in the placebo arm. Orthostatic hypotension was not associated with any falls or other symptoms. One fall was notified in a patient of the clonidine arm but unrelated to orthostatic hypotension. One asymptomatic bradycardia was detected during follow-up in the clonidine group (between 50 and 60 bpm). Sleepiness was reported in one patient in the clonidine arm and two patients in the placebo arm.

**Table 3:** Adverse events related to the treatment

	Placebo group	Clonidine group
Fatigue	3	2
Asmptomatic bradycardia < 60 bpm but > 50 bpm	0	1
Restless legs syndrome	0	1
Fall (without orthostatic hypotension)	0	1
Nausea	0	1
Orthostatic hypotension	2	3
Sleepiness	2	1

## Discussion

This study found that clonidine was well tolerated by PD patients, and the results indicate that a phase 3 trial should be conducted. Although the rate of success was not significantly different between groups, patients receiving clonidine had a greater and faster, reduction of the total QUIP-RS score at 8 weeks; it is of note that this phase 2b study was not calibrated to conclude on clonidine efficacy but on the conduct a phase 3 study. Of course, the pathophysiology of ICD in PD being still controversial and complex, we cannot exclude that targeting only the noradrenergic system could be insufficient to suppress ICD [2]. Furthermore, it is worth mentioning the importance of the placebo effect which has been reported to be strong in PD [34]. This suggests also that patients can modify their addictive behaviour thanks to a close follow-up with regular neuropsychologists interviews, neurological follow-up and support. It would be interesting to evaluate more precisely the impact of psychological intervention for the treatment of ICDs [35]. In terms of tolerance, induced orthostatic hypotension was not an issue and was found in three patients in the clonidine arm and two in the placebo arm, despite the reduction of peripheral vascular resistance induced by clonidine. Moreover, the Epworth score at 8 weeks was slightly better in the clonidine group and only one case of sleepiness was reported in the clonidine arm (2 in the placebo group), which was another potential limitation for the use of this drug in PD patients. Finally, PDQ39 was better in the clonidine group after 8 weeks of treatment.

Despite the randomisation, an imbalance was observed between groups for several variables, which, in turn, could have affected the results. Indeed, patients in the clonidine group had higher QUIP-RS total scores at baseline, meaning that they experienced more severe ICD. Moreover, patients in the clonidine groups were less systematically on agonist dopaminergic (78.9% vs 100% in the placebo group), which could, again, reflect more important ICD persisting despite the discontinuation of this treatment. Altogether, we cannot rule out that patients in the clonidine group may present more severe and more difficult to tackle ICD. It was however hard, because of the difficulty of recruiting these patients, to perfectly match them based on ICD score for this early-phase study. Although this could have affected our findings, we hypothesize that the differences of trends in terms of QUP-RS score reduction could have been more pronounced with better matching, which reinforces the need for a larger study to smooth out inter-group differences. In addition, with our mixed-effects model considering this difference, the difference in slopes remains statistically significant between the arms at the 10% alpha threshold (with *p* = 0.06).

The precise mechanisms of action of clonidine remain to be elucidated. It could, however, impact ICDs via the modification of noradrenergic transmission affecting, in turn, the inhibitory role of the prefrontal cortex; this lack of inhibitory and executive top-down control is one of the mechanisms of behavioural addictions in PD, in combination with aberrant motivation and reward circuits activations [36]. In theory, activation of presynaptic α2-adrenoceptors results in the inhibition of neural release of NA, but a high dose of clonidine has been reported to engage post-synaptic receptors leading to an enhancement of noradrenergic transmission [18]. Herein, the dose used is considered as high suggesting that the observed trend might be related to increased noradrenergic tonus, which fits well the positive effect of atomoxetine, a NA transporter antagonist increasing noradrenergic release, on response inhibition during a stop signal task in PD [37]. Interestingly, a higher dose is used in Tourette syndrome [25], which could suggest a higher dose of clonidine in future studies on ICDs in PD, but it seems more appropriate that a longer treatment duration be explored to maximize clonidine efficacy. This is supported herein by the clonidine-induced reduction of the total QUIP-RS score that was more pronounced after 8 than 4 weeks, suggesting a progressive action. In addition, as suggested in a recent clinical trial of atomoxetine in PD patients with apathy, the effect of atomoxetine appears dependent on locus coeruleus integrity [38] and it would therefore be useful to include this aspect using imaging approaches (MRI or PET) in future studies investigating clonidine in such patients. To conclude, this phase 2b study paves the way for a large phase 3 study with a longer treatment duration to demonstrate or rule out an effect of clonidine on ICDs in PD.

### Supplementary Information

Below is the link to the electronic supplementary material.

Supplementary file1 (DOCX 20 KB)

Supplementary file2 (DOCX 14 KB)
