# Supplementary material for: Efficacy and safety of clonidine for the treatment of impulse control disorder in Parkinson’s disease: a multicenter, parallel, randomised, double-blind, Phase 2b Clinical trial
Source: J Neurol. 2023 Jun 20;270(10):4851–9. doi: 10.1007/s00415-023-11814-y (PMC10511565; doi:10.1007/s00415-023-11814-y)
Supplement: Supplementary file 1 — Supplementary file1 (DOCX 20 KB) [file 415_2023_11814_MOESM1_ESM.docx]

Annex 1 -About the mixed model

Random effects :

| Coefficient | Estimation | Interpretation |
| --- | --- | --- |
| Random intercept | 9.9 | 95 % of within-group-patients have a baseline QUIP of +/-19.4 around the mean group baseline QUIP |
| Random slope | 5,5 | 95 % of within-group-patients have a slope of +/- 10.8 around the mean slope group. |
| Covariance random intercept – random slope | -34.4 | Negative covariance says that a patient with higher baseline QUIP would have a higher reduction of QUIP. |


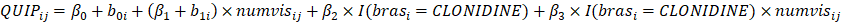


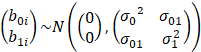


*Patient i, visit j (baseline , week 4, week 8)*

*Numvis_ij_ = j^th^ visit of the i^th^ patient*

*Bras_i_ = treatment group of the i^th^ patient*
