# Supplementary material for: Efficacy and safety of clonidine for the treatment of impulse control disorder in Parkinson’s disease: a multicenter, parallel, randomised, double-blind, Phase 2b Clinical trial
Source: J Neurol. 2023 Jun 20;270(10):4851–9. doi: 10.1007/s00415-023-11814-y (PMC10511565; doi:10.1007/s00415-023-11814-y)
Supplement: Supplementary file 2 — Supplementary file2 (DOCX 14 KB) [file 415_2023_11814_MOESM2_ESM.docx]

**prohibited treatment**

diuretic

vasodiltator

beta blockers

calcium antagonist

Angiotensin Converting Enzyme Inhibitors

Angiotensin II receptor antagonists

digoxine

mianserin

mirtazapine

venlafaxine

milnacipran

duloxetine

tramadol

methylphenidate

amitriptyline

clomipramine

modafinil

baclofene

neuroleptic
